# Low Dietary Diversity and Intake of Animal Source Foods among School Aged Children in Libo Kemkem and Fogera Districts, Ethiopia

**DOI:** 10.1371/journal.pone.0133435

**Published:** 2015-07-23

**Authors:** Zaida Herrador, Jesus Perez-Formigo, Luis Sordo, Endalamaw Gadisa, Javier Moreno, Agustin Benito, Abraham Aseffa, Estefania Custodio

**Affiliations:** 1 National Centre of Tropical Medicine, Instituto de Salud Carlos III (ISCIII), Madrid, Spain; 2 Tropical Diseases Research Network (RICET in Spanish), Madrid, Spain; 3 National Centre of Epidemiology, ISCIII, Madrid, Spain; 4 Spanish Field Epidemiology Training Program (PEAC, in Spanish), Madrid, Spain; 5 Department of Preventive Medicine and Public Health, Faculty of Medicine, Complutense University, Madrid, Spain; 6 Network Biomedical Research Centers, Epidemiology and Public Health (CIBERESP), Madrid, Spain; 7 Armauer Hansen Research Institute, Addis Ababa, Ethiopia; 8 National Centre of Microbiology, ISCIII, Madrid, Spain; 9 Monitoring Agricultural Resources Unit-H04, Institute for Environment and Sustainability, European Commission Joint Research Center, Ispra, Italy; TNO, NETHERLANDS

## Abstract

**Background:**

A low dietary diversity score (DDS) and low consumption of food from animal sources (ASF) are among the factors related to malnutrition in school-aged children living in Libo Kemkem and Fogera (Ethiopia).

**Objectives:**

This study aimed to identify associated determinants for low dietary diversity and lack of consumption of ASF.

**Methods:**

In 2009, a cross-sectional survey was carried out in May, at the end of the lean season. Socio-demographic characteristics and diet habits were collected from 886 school-aged children. Additionally, 516 children from rural sites were followed up in the post-harvest season, in December of the same year. Bivariate and multivariable statistical methods were employed to assess low DDS and ASF intake and their association with different factors.

**Results:**

Up to 80% and 60% of school-aged children living in rural and urban sites, respectively, ate ≤ 3 food groups the day before the survey. The percentage of children consuming ASF was significantly higher in urban settings (64% *vs* 18%). In the rural areas, if the head of the household was male (OR: 1.91; 95%CI: 1.00-3.65) and older than 40 years (OR: 1.56; 95%CI: 1.02-2.38) the child had a lower DDS in the lean season, while differences by socioeconomic indexes were observed in the post-harvest season. Males took more ASF than females in rural settings (OR: 1.73; 95%CI: 1.14-2.62) and differences by socioeconomic indexes were observed in both settings in the lean season, though not in post-harvest survey.

**Conclusions:**

The findings of this study revealed that the diet among school-aged children in Libo Kemkem and Fogera districts lacked diversity, and that the intake of foods from animal sources was low, especially among rural girls. To effectively tackle malnutrition, dietary diversification strategies oriented to the local needs are recommended.

## Background

Food consumption is one of the immediate causes of children malnutrition according to the UNICEF casual framework [1]. Food consumption may be affected by food availability, stability of the food supply, food access, and food utilization, the four pillars of food security [2], as well as by physiological and health status, cultural patterns, perceptions and societal conventions, among others [3].

The global number of hungry people declined by 132 million between 1990–92 and 2010–12, from 23.2 percent to 14.9 percent in developing countries, being the decline between 1990 and 2007 more pronounced than previously expected. However, since 2007–2008, global progress in reducing hunger has slowed and leveled off, and according to the latest estimates from the Food and Agriculture Organization of the United Nations (FAO), still in 2012 more than 800 million people were undernourished, e.g. with insufficient food for an active and healthy life[4]. The greatest food security challenges overall remain in sub-Saharan Africa, which has seen particularly slow progress in improving access to food, with slow-moving income growth, high poverty rates and poor infrastructure, which hampers physical and distributional access [5]. Moreover, food availability remains low, even though energy and protein supplies have improved [6].

In Ethiopia, the primary underlying causes of undernourishment are agricultural market dysfunctions, rapid population growth, poor infrastructure, decline of the per capita food grain production and other institutional and organizational failures [7]. Ethiopia is mainly dependent on the agricultural sector; about 75% of the population is engaged in agriculture, especially in subsistence and rain-fed farming and livestock production [8]. Food availability, supply and access are strongly affected by seasonality; many households are only able to produce sufficient food to meet their food requirements for less than six months of the year, facing acute food shortages during the hunger season [9,10].Moreover, even in adequate food producing regions of Ethiopia (such as Amhara), high prevalence rates of stunting have been reported, thus indicating that while food security is necessary, it is not the only determining factor for ensuring nutrition security [10,11].

Food consumption patterns across Ethiopia are diverse, and the food basket consists of a wide variety of grains and other staples, which change widely according to differences in agro-ecology, socioeconomic levels, and livelihood strategies [12]. As in many other traditional societies, dietary preferences and consumption patterns are also influenced by cultural values and traditions and may not necessarily reflect availability or the nutritional quality of specific food items [13]. Moreover, given dependence on own production, food grain consumption varies at different times of the year [12]. Inequalities in dietary patterns among urban and rural areas exist too [14,15], and may be due to the fact that the population residing in rural areas have lower level of income [16]. Furthermore, people residing in rural areas may face some extra challenges such as social isolation, droughts and limited access to transportation, market, installations and health services [17].

Since the 90s researchers around the world have been promulgating the variety of foods in the diet, the underlying principle being that variety will ensure an adequate intake of essential nutrients and hence promote good health [18]. Dietary diversity (DD) indicators have been used as proxies of quality food consumption and food security [19] for various reasons. Dietary diversity indicators may capture consumption of both macro and micronutrients, or a more balanced diet in the general sense without the need of measuring the quantity of food consumed, which may turn difficult in certain contexts. Furthermore there are economic theories of demand as well as psychological ones suggesting that individuals will diversify into higher value micronutrient rich foods (such as meats, fish, dairy products, etc.) only when they have satisfied their basic calorie needs [20]. Chronic malnutrition and micronutrients deficiencies in school-aged children living in Fogera and Libo Kemkem have been previously associated with a low DD and the lack of ASF consumption, together with other socio demographic factors [21,22]. In children, low DD is typically expressed as a monotonous diet that is mainly based on low energy and nutrient density foods, such as cereals or tubers, and too low in nutrient rich foods, like animal source foods (ASF) [23].

A better knowledge of the dietary habits of this population and its related factors is needed in guiding the design of interventions to improve food consumption and dietary diversity beyond the specific supplementation programs. Therefore, the aim of this study was to provide an in-depth description of the food consumption and dietary diversity among the school age children in in the study area.

## Methods

### Study area and population

The study was carried out in May and December 2009, during the lean and the post-harvest season, respectively, in the districts (*woredas*) of Libo Kemkem and Fogera, in Tana Zuria Livelihood Zone (within Amhara regional state, Northwest of Ethiopia). This zone is amongst the two or three most food self-sufficient livelihood zones in Amhara [10]. Libo Kemkem and Fogera *woredas* are located at an altitude of 2,000 m above sea level. According to the 2009 census, the population was 198,374 and 226,595 for Libo Kemkem and Fogera, respectively. The population is scattered across the zone in somewhat concentrated dense settlements. Temperatures are relatively high, but rainfall is unusually abundant at 1,173 mm per annum as the long-term mean.

Agriculture activities are dependent on a single rainy season (from June to September). Land preparation begins in February and continues until May when long cycle crops are sown. A second land preparation phase for the cultivation of short-cycle crops occurs during the rainy season in August. The consumption year begins in October, lasting until January. The major hazards to crop production are pests and occasional flooding in some districts [24].

Livestock holdings in sheep and cattle are modest, but livestock and butter sales make a substantial compliment to the dominant crop sales. The livestock production season begins with both cattle and small stock births in June. The main lactation period lasts for 5 months, until November. The peak trading season is during the religious festivals in January, April and September; and in October and November when plowing activities are complete [10].

Market access is good largely due to the good road network, helping the strong outflow of products from the zone. Overall, trading activities peak during the post-harvest season, starting from October-November, in a relatively good year by local standards. The season of food short supply, in most cases, is just before the harvest, when previous year’s grain stores are nearly finished and market prices are high [10].

### Study design

This study was part of a UBS Optimus Foundation funded project called Visceral Leishmaniasis (VL) and Malnutrition in Amhara State, Ethiopia. Other methodological aspects have already been published in previous papers [22,25,26].

Sample size was calculated according to previous estimates of malnutrition for children < 5 years old in the area and taking into account a design effect of 2, corresponding to the complex design. Sampling was carried out by multistage cluster survey. Primary sampling units were sub-districts (*kebeles*). Secondary sampling units were randomly selected villages (*gotts*) in each of the selected sub-districts. Third sampling units were randomly selected households in each of the villages. All children with reported age between 4 and 15 years living in the household at the time of the survey were included in the study. 889 children aged 4 to 15 years were finally recruited. Out of them, 514 school-aged children living in rural areas (57.8%) were randomly selected for follow up in the post-harvest season, in December 2009. Study variables did not differ between the complete rural sample and those who were followed-up at the 0.05 probability level (S1 Table).

### Data collection and management

The survey consisted of three pre-tested questionnaires (individual, household and community), all translated into Amharic, the local language, and administered by trained medical personnel (nurses and health officers). The community questionnaire was addressed to the community leader and it compiled information related to community assets and services. The household questionnaire was administered to the caretaker of the study children and consisted of information on household socio-demographic characteristics and house construction material and assets (land and cultivation, domestic animals assets, etc.). Finally, the individual questionnaire, also administered to the caretaker, targeted behavioral and biological characteristics, and was also comprised of a 24-hour dietary recall.

Three area-based socioeconomic indexes were calculated by performing Principal Component Analysis (PCA) [27,28]: the socio-economic index (SES), the socio-educative index (SED) and the community endowment index (CEI). Separate SES indexes were constructed for urban and rural settings, using different lists of assets, due to fundamental differences in infrastructure and lifestyle between urban and rural areas [27]. The variables included in each of the indices are described in the S2 Table.

A low dietary diversity score (DDS) and the consumption of any food from animal sources were the main outcomes of interest. Based on the Food and Agriculture Organization of the United Nations (FAO)/ Food and Nutrition Technical Assistance (FANTA) Guidelines for measuring Household and Individual Dietary Diversity [29], the dietary data collected through the 24-hour diet recall were computed into 9 food groups: cereals, roots and tubers; vitamin-A-rich fruits and vegetables; other fruit; other vegetables; legumes and nuts; meat, poultry and fish; fats and oils; dairy; and eggs. The Dietary Diversity Score (DDS) was calculated by summing the number of unique food groups. Accordingly, the level of diet diversity was computed out of the score of 9. The Dietary Diversity Score is based on the computation of the different food groups consumed but it does not require to know the quantity, which was not measured in this study due to methodological restrictions.

To assess the consumption of animal source foods (ASF), a new variable was computed by considering the consumption of any of the following three food groups: meat, poultry and fish, dairy, and eggs.

### Statistical analysis

Frequencies and percentages were used to summarize data and to explore the differences among rural and urban communities. These differences were assessed by Student's t-test and χ^2^ tests for continuous and categorical variables, respectively. The socio-economic indexes and the DDS were used to categorize participants into tertiles representing the lower, middle, and upper one-thirds of every index/score in the study population.

Bivariate analyses to assess the associated factors with low tertiles DDS and with the consumption of any food from animal sources were performed, both stratified by setting. All variables associated with each of the outcomes at the p <0.10 level were included in the multivariable analysis. Logistic regression models were obtained by using a manual backward stepwise procedure. Same analysis was also performed in post-harvest season for those living in rural sites. P-values less than or equal to 0.05 were considered statistically significant. The unadjusted and adjusted odds ratio (uOR and aOR, respectively) with the 95% confidence intervals (95% CI) were computed. The goodness of fit was assessed using Hosmer-Lemeshow statistic. Data analysis was performed using SPSS version 18.0 (SPSS Inc., Chicago, Illinois, USA).

### Ethical considerations

The study was approved by the ethical review boards of the Instituto de Salud Carlos III (N° PI:355, obtained in July 15th, 2008) and of the Armauer Hansen Research Institute (P:015/08,obtained in November 5th, 2008), and by the Ethiopian National Ethical Review Committee (RDHE/57-86/2009, obtained in February 11th 2009). Support letters were obtained from the Amhara State and health bureaus. All parents/guardians gave written informed consent before enrollment of their children in the study.

## Results

### Description of the sample

The study included a total of 886 children aged 4 to 15 years, of which 462 (52.0%) were males. Around 80% lived in the rural setting and 78.9% referred a low tertile DDS, more frequently in rural than in urban communities (83.6% and 60.1% respectively; p<0.001). Individual and household characteristics, as well as socioeconomic indexes are summarized in Table 1, disaggregated by rural and urban strata. There are statistically significant differences between children living in rural and urban settings for all items except for the sex and the age of the child (Table 1).

**Table 1:** Comparison of individual and household characteristics in school-aged children by rural and urban setting, Libo Kemkem and Fogera districts, May 2009.

VARIABLES		Total (889)		Rural (711)		Urban(178)	
		n	*(%)*	n	*(%)*	n	*(%)*
**INDIVIDUAL CHARACTERISTICS**							
**Sex (girls)**		427	*48*	339	*47*.*7*	88	*49*.*4*
**Age (> = 10 years)**		340	*38*.*2*	275	*38*.*7*	65	*36*.*5*
**DDS high tertile (>4 groups)**		44	*5*	28	*4*.*0*	16	9.0*
**DDS low tertile (≤ 3 groups)**		699	*78*.*9*	592	*83*.*6*	107	60.1**
**HOUSEHOLD CHARACTERISTICS**							
**Sex of household's head (female)**		127	*14*.*3*	52	*7*.*3*	75	42.1**
**Age of household's head (>40 years)**		416	*47*.*2*	351	*49*.*7*	65	36.9*
**Religion of household's head (orthodox)**		853	*96*	706	*99*.*3*	147	82.6**
**Occupation of household's head (unqualified)**		98	*11*	709	*99*.*7*	82	46.1**
**School years of household´s head (< = 4 years)**		693	*78*	594	*83*.*5*	99	55.6**
**School years of person in charge of food preparation (< = 4 years)**		817	*91*.*9*	702	*98*.*7*	115	64.6**
**Number of children in the household (> 3 children)**		201	*22*.*6*	192	*27*	9	5.1**
**Number of people in household (> 6 people)**		363	*40*.*9*	326	*46*	37	20.8**
**Have domestic animals (yes)**		750	*84*.*4*	685	*96*.*3*	65	36.5**
**Own land (yes)**		712	*80*.*1*	694	*97*.*6*	18	10.1**
**SOCIO-ECONOMIC INDEXES**							
**Socio-Economic Index**	**Low**	289	*32*.*5*	238	*33*.*5*	51	*28*.*7*
	**Medium**	283	*47*.*2*	236	*36*.*2*	47	26.4*
	**High**	317	*35*.*7*	237	*33*.*3*	80	44.9*
**Socio-Educative Index**	**Low**	466	*52*.*4*	390	*54*.*9*	76	*42*.*7*
	**Medium**	190	*21*.*4*	164	*23*.*1*	26	14.6**
	**High**	233	*26*.*2*	157	*22*.*1*	76	*42*.*7*
**Community Endowment Index**	**Low**	285	*32*.*1*	247	*34*.*7*	38	*21*.*3*
	**Medium**	283	*31*.*8*	177	*24*.*9*	106	*59*.*6* **
	**High**	321	*36*.*1*	287	*40*.*4*	34	*19*.*1*

DDS: diet diversity score

*p<0.05

**p<0.001.

### Food consumption

**Fig 1:**
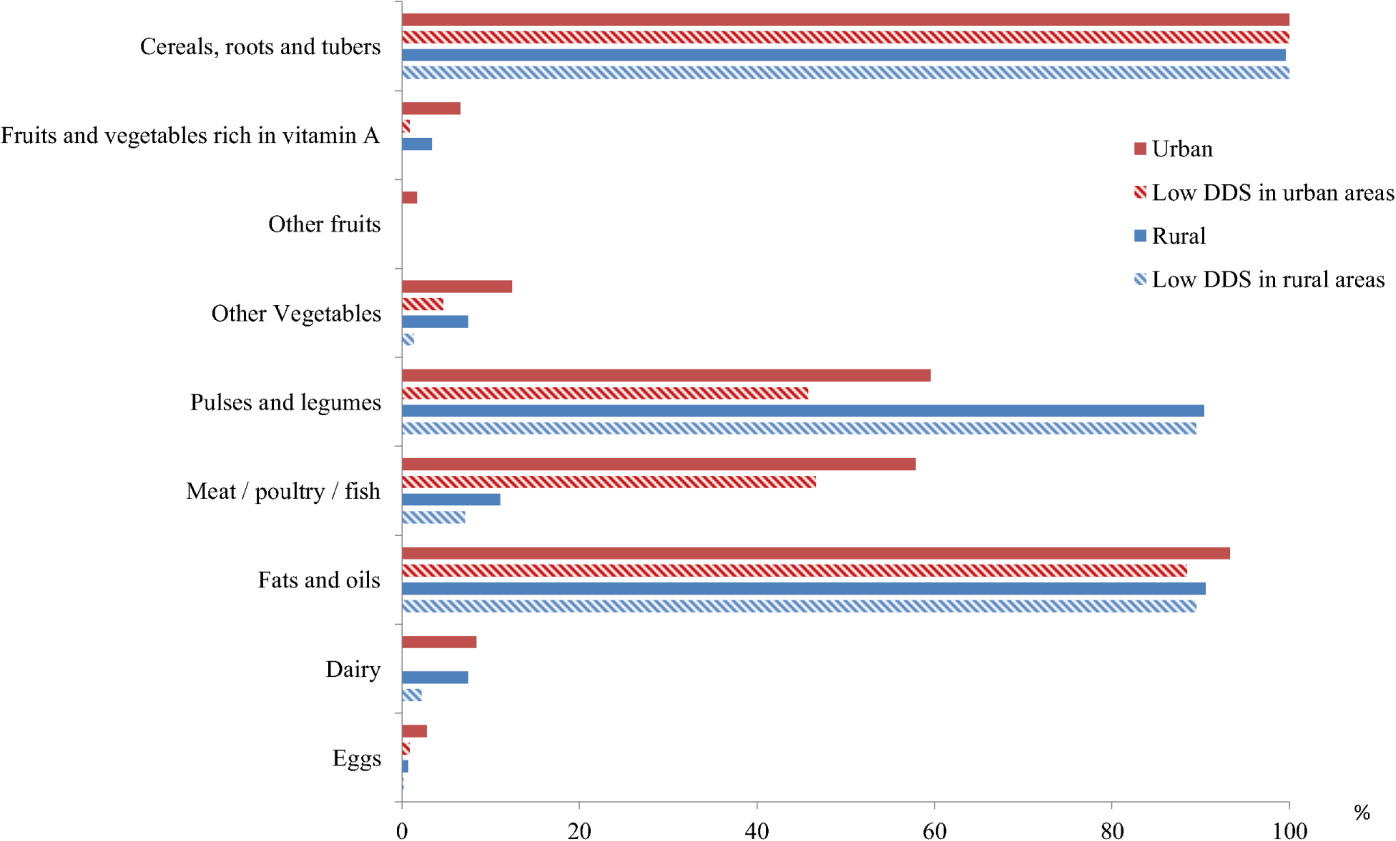
Percentage of overall school-aged children and those belonging to the low DDS tertile who consume each food group by setting, May 2009, Libo Kemkem and Fogera, Ethiopia.

The diet of the study population contained mostly basic staples, legumes, pulses and oil. Specifically, the diet in rural areas was mainly based on cereal, roots and tubers (99.6%) and pulses and legumes (90.4) while children living in urban settings had a more diversified diet (Fig 1, Table 2). Less than 12% and 8% of the interviewees in rural sites reported intake of meat or dairy products in the previous day, respectively. The percentage of children consuming meat was significantly higher in urban settings (57.9%, p<0.001). Pulses and legumes were more frequently consumed in rural areas (p<0.001) while other vegetables and eggs intake were more common among those children living in urban areas (p = 0.035 and p = 0.017, respectively).

**Table 2:** Comparison of food groups and food items consumed in urban and rural settings in May 2009 and in rural setting in December 2009, Libo Kemkem and Fogera districts, Ethiopia.

Food groups and food items	Rural (n = 708) May 2009		Urban (n = 178) May 2009		Rural (n = 516) Dec 2009 (versus Rural in May)	
	n	*%*	n	*%*	n	*%*
**Group 1. Cereals. roots and tubers**	**708**	***99*.*6***	**178**	***100***	**515**	***99*.*8***
Teff*	464	*65*.*5*	160	89.9**	375	*72*.*8*
Finger Millet	470	*66*.*4*	47	26.4**	175	34**
Rice	262	*37*	44	*24*.*7* *	242	47**
Maize	235	*33*.*1*	36	*20*.*2* *	184	*35*.*7*
*Malat* *	104	*14*.*6*	10	*5*.*6* *	72	*14*
Wheat	69	*9*.*8*	46	25.8**	11	2.1**
*Kucho* *	58	*8*.*2*	26	*14*.*6* *	29	*5*.*6*
Potato	17	*2*.*4*	10	*5*.*6* *	8	*1*.*6*
*Bule* *	40	*5*.*6*	40	*22*.*5* *	-	*-*
Bread	1	*0*.*1*	1	*0*.*6*	12	2.3**
Shorgum	24	*3*.*4*	-	*-*	1	0.2**
**Group 2. Fruits and vegetables rich in vitamin A**	**24**	***3*.*4***	**11**	***6*.*2***	**1**	0.2**
**Group 3. Other fruits**	**-**	*** ***	**3**	***1*.*7***	**-**	**-**
**Group 4. Other Vegetables**	**53**	***7*.*5***	**22**	***12*.*4*** *	**93**	18**
Red Pepper	21	*2*.*9*	5	*2*.*8*	71	13.8**
Tomato	13	*1*.*8*	13	7.3**	17	*3*.*3*
Green Pepper	12	*1*.*7*	1	*0*.*6*	10	*1*.*9*
Cabbage	2	*3*.*8*	3	*13*.*6* *	**-**	**-**
**Group 5. Pulses and legumes**	**643**	***90*.*4***	**106**	57.6**	**486**	*94*.2*
Chick-peas	522	*73*.*4*	51	28.7**	423	82**
Beans	33	*4*.*7*	42	23.6**	39	*7*.*6*
Guaya	9	*5*.*1*	254	35.7**	106	*20*.5*
Lentils	15	*2*.*1*	4	*2*.*2*	38	*7*.*4*
**Group 6. Meat / poultry / fish**	**79**	***11*.*1***	**103**	57.9**	**6**	1.2**
Goat	26	*3*.*7*	53	29.8**	**-**	**-**
Beef	36	*5*.*1*	17	*9*.*6* *	**-**	**-**
Chicken	8	*1*.*1*	15	8.4**	**-**	**-**
Sheep	5	*0*.*7*	15	14.6**	2	*0*.*4*
**Group 7. Fats and oils**	**644**	***90*.*6***	**166**	***93*.*3***	**354**	68.6**
**Group 8. Dairy**	**53**	***7*.*5***	**15**	***8*.*4***	**97**	18.8**
Milk	44	*6*.*2*	10	*5*.*6*	86	16.7**
Butter	2	*0*.*3*	5	*2*.*8* *	7	*1*.*4*
*Ayib* *	1	*0*.*1*	**-**	**-**	1	*0*.*2*
Cow Milk	3	*0*.*4*	**-**	**-**	12	*2*.*3* *
*Ergo* *	**-**	**-**	2	*1*.*1*	2	*0*.*4*
**Group 9. Eggs**	**5**	***0*.*7***	**5**	2.8*	**-**	**-**
**Any food from animal source#**	**129**	***18*.*1***	**114**	64**	**102**	***11*.*5***

* Typical Ethiopian food items. ***Teff***: a species of lovegrass native to the northern Ethiopian Highlands and Eritrean Highlands of the Horn of Africa. ***Malat***: Ethiopian cereal. ***Kucho***: flour made from sycamore root (with fiber). ***Bule***: flour made from sycamore bark (without fiber). ***Ayib***: soft Ethiopian fresh cheese similar to cottage cheese. ***Ergo***: traditional Ethiopian fermented milk.

# Consumption of any of the following three food groups: meat, poultry and fish, dairy and eggs.

Dec: December.

*p<0.05

**p<0.001.

Overall, *teff*, finger millet and chickpeas were the most common eaten cereals and legumes, though differences were also observed by setting. For instance, finger millet, rice, maize and chick-peas were more frequently consumed by school-aged children living in rural areas while *teff (Eragrostis tef)*, wheat, *kotcho (a pan made of flour made from the bark and root of Ensete ventricosum)*, potato, *bula*, (*a porridge of flour made from the bark and root of Ensete ventricosum)* beans (*Vicia faba*) and *guaya* (*Lathyrus sativus*) were more prevalent in the urban diet. No child reported consumption of fish during the day before the survey in any of the settings (Table 2).

Food groups´ intake also varied by season in the rural environment. Cereals, roots and tubers remained the most consumed food group in the post-harvest season, though significant changes were observed concerning other food groups. While the intake of meat and fats and oils was significantly lower in this post-harvest season (p<0.001), the consumption of pulses and legumes, other vegetables (non-rich in vitamin A), and dairy products was higher (90.4% versus 7.5% and 7.5% versus 94.2%, 18% and 18.8%, respectively). Also, seasonal differences in the type of food items included in each food group were found (Table 2).

### Dietary diversity and related factors

Up to 80% and 60% of school-aged children living in rural and urban sites, respectively, ate 3 or less food groups the day before the survey. Cereals, roots and tubers were consumed by all school-aged children with low DDS, regardless the setting, whereas pulses and legumes contributed especially to the diet of children with low DDS living in rural areas. Children with low DDS from rural areas did not consume any fruits or vegetables while those from urban sites did not take any dairy (Fig 1).

In the rural settings, school-aged children had a lower DDS if the head of the household (HH) was male and older than 40 years than if the HH was female and younger than 40 years, (OR: 1.91; 95%CI: 1.00–3.65; and OR: 1.56; 95%CI: 1.02–2.38, respectively). In the post-harvest survey, associations between low tertile DDS and SES and SED were found. Children with low SES had a lower DDS than those with higher SES level (86.7% *vs* 79.3% in high SES level, p<0.001). On the contrary, having a low DDS was more frequent in those children belonging to families with higher SED compared to the lowest class (OR: 2.20; 95%CI: 1.13–4.26).

The only variable associated with low DDS in the urban setting was SED; a low DDS was almost three times less common in children whose family had a high SED compared to those with the lowest SED level (OR: 0.36; 95%CI: 0.19–0.71, Table 3).

**Table 3:** Low tertile Dietary Diversity Score (≤3 food groups) associated factors among school-aged children by setting, Libo Kemkem and Fogera districts, May 2009.

VARIABLES		Rural May 2009 (n = 711)				Urban May 2009 (n = 178)				Rural Dec 2009 (n = 516)			
		LDDS		uOR (95%)	aOR (95%)	LDDS		uOR (95%)	aOR (95%)	LDDS		uOR (95%)	aOR (95%)
		n	%			n	%			n	%		
**INDIVIDUAL CHARACTERISTICS**													
**Sex**	**Female**	337	*83*.*7*			88	*64*.*7*			256	*85*.*2*		
	**Male**	371	*83*.*0*	0.91 (0.61–1.36)	**-**	90	*55*.*6*	0.68 (0.37–1.24)	**-**	259	*81*.*5*	0.77 (0.48–1.22)	**-**
**Age**	**<10 year**	435	*82*.*5*			113	*61*.*1*			315	*83*.*2*		
	**> = 10 year**	273	*85*.*3*	1.23 (0.81–1.87)	**-**	65	*58*.*5*	0.90 (0.48–1.67)	**-**	200	*83*.*5*	1.02 (0.64–1.65)	**-**
**HOUSEHOLD CHARACTERISTICS**													
**Sex Head Household**	**Female**	52	*71*.*2*			75	*69*.*3*			25	*67*.*6*		
	**Male**	656	*84*.*6*	2.23 (1.18–4.21) **	1.91 (1.00–3.65)**	103	*53*.*4*	0.51 (0.270.95) **	0.63 (0.32–1.22)	404	*84*.*5*	2.62 (1.26–5.45)**	2.74 (1.27–5.90)**
**Age Head Household**	**< = 40 years**	353	*79*.*9*			111	*57*.*7*			215	*81*.*1*		
	**> 40 years**	350	*87*.*1*	1.58 (1.04–2.39) **	1.56 (1.02–2.38)**	65	*63*.*1*	1.26 (0.67–2.35)		209	*85*.*3*	1.35 (0.85–2.16)	**-**
**Religion Head Household**	**Orthodox**	703	*83*.*6*			147	*59*.*9*		**-**	425	*83*.*3*		
	**Others**	5	*80*	0.78 (0.09–7.06)	**-**	31	*61*.*3*	1.06 (0.48–2.35)		4	*80*	0.80 (0.09–7.25)	**-**
**Number of people in household**	**< = 6 person**	381	*81*.*9*			141	*63*.*8*		**-**	233	*81*.*8*		
	**> 6 person**	325	*85*.*5*	1.31 (0.87–1.96)	**-**	37	*45*.*9*	0.48 (0.23–1.00)**	0.73 (0.33–1.62)	195	*85*.*2*	1.28 (0.80–2.05)	**-**
**Number of children in household**	**< = 3 children**	517	*82*.*4*			168	*61*.*3*			306	*82*.*9*		
	**> 3 children**	191	*86*.*9*	1.42 (0.89–2.29)*	1.28 (0.77–2.03)	9	*44*.*4*	0.51 (0.13–1.95)	**-**	123	*84*.*2*	1.10 (0.65–1.85)	**-**
**SOCIO-ECONOMIC INDEXES**													
**Socio-Economic Index**	**Low**	236	*84*.*3*	1		51	*70*.*6*	1	1	137	*86*.*7*	1	1
	**Medium**	235	*83*	0.91 (0.56–1.48)	**-**	47	*66*	0.81 (0.34–1.89)	0.89 (0.37–2.15)	150	*84*.*3*	0.82 (0.45–1.51)	0.75 (0.40–1.41)
	**High**	237	*83*.*5*	0.94 (0.58–1.54)	**-**	80	*50*	0.42 (0.20–0.88)**	0.61 (0.27–1.38)	142	*79*.*3*	0.59 (0.33–1.06)*	0.46 (0.25–0.85)**
**Socio-Educative Index**	**Low**	388	*82*	1		76	*72*.*4*	1	1	225	*80*.*1*	1	1
	**Medium**	163	*84*	1.16 (0.71–1.90)	**-**	26	*57*.*7*	0.52 (0.21–1.32)	0.61 (0.24–1.60)	94	*84*.*7*	1.38 (0.76–2.49)	1.28 (0.70–2.36)
	**High**	157	*87*.*3*	1.51 (0.88–2.57)	**-**	76	*48*.*7*	0.36 (0.19–0.71)**	0.41 (0.20–0.83)**	110	*89*.*4*	2.11 (1.11–4.01)**	2.20 (1.13–4.26)**
**Community Endowment Index**	**Low**	247	*81*.*4*	1		38	*55*.*3*	1		173	*81*.*6*	1	
	**Medium**	176	*84*.*1*	1.21 (0.72–2.03)	**-**	106	*59*.*4*	1.19 (0.56–2.51)	**-**	87	*82*.*9*	1.09 (0.59–2.02)	**-**
	**High**	285	*85*.*3*	1.32 (0.84–2.09)	**-**	34	*67*.*6*	1.69 (0.65–4.43)	**-**	169	*85*.*4*	1.31 (0.78–2.22)	**-**
**Goodness of fit**		**Nagelkerke R Square: 0.027; Hosmer and Lemeshow Test p>0.05**				**Nagelkerke R Square: 0.099; Hosmer and Lemeshow Test p>0.05**				**Nagelkerke R Square: 0.099; Hosmer and Lemeshow Test p>0.05**			

* p<0.10

**p<0.05. LDDS: low dietary diversity index. uOR: unadjusted odds ratio. aOR: adjusted odds ratio.

### Consumption of animal source foods and related factors

The percentage of children consuming ASF was significantly higher in the urban settings compared to the rural areas (64% *vs*. 18.1%, respectively; p<0.001) (Table 1). In the survey conducted in the lean season in rural areas, being male was associated with higher intake of ASF (aOR: 1.73; 95%CI: 1.14–2.62), as well as having a medium or high SES score. Conversely, living in a household with a head of the household 40 years or older and with more than 3 children, decreased the likelihood of having consumed ASF the day before (aOR: 0.46; 95%CI: 0.29–0.72) and (aOR: 0.47; 95%CI: 0.27–0.83) respectively. Also, higher SED and CEI scores were associated with less frequent consumption of ASF in this sample population. In the post-harvest survey conducted in rural sites, only the age of the head of the household remained inversely associated with the consumption of animal source foods (Table 4).

**Table 4:** Consumption of animal source foods associated factors among school-aged children by rural and urban setting in Libo Kemkem and Fogera districts, May-December 2009.

VARIABLES		Rural May 2009 (n = 711)				Urban May 2009 (n = 178)				Rural Dec 2009 (n = 516)			
		Any food from animal source		uOR (95%)	aOR (95%)	Any food from animal source		uOR (95%)	aOR (95%)	Any food from animal source		uOR (95%)	aOR (95%)
		n	%			n	%			n	%		
**INDIVIDUAL CHARACTERISTICS**													
**Sex**	**Female**	51	*15*			57	*64*.*8*			44	*17*.*2*		
** **	**Male**	78	*21*	1.50 (1.02–2.21)**	1.73 (1.14–2.62)**	57	*63*.*3*	0.94 (0.51–1.73)	**-**	58	*22*.*3*	1.38 (0.89–2.14)	**-**
**Age**	**<10 year**	81	*18*.*6*			74	*65*.*5*			71	*21*.*5*		
** **	**> = 10 year**	48	*17*.*5*	0.93 (0.63–1.37)	**-**	40	*61*.*5*	0.84 (0.45–1.59)	**-**	31	*16*.*8*	0.74 (0.46–1.18)	**-**
**HOUSEHOLD CHARACTERISTICS**													
**Sex Head Household**	**Female**	17	*32*.*7*			77	*74*.*8*			7	*18*.*9*		
	**Male**	112	*17*	0.42 (0.23–0.78)**	0.69 (0.35–1.36)	37	*49*.*3*	3.04 (1.61–5.74)**	2.09 (0.91–4.80)*	95	*19*.*8*	1.06 (0.45–2.49)	**-**
**Age Head Household**	**< = 40 years**	79	*22*.*3*			72	*64*.*9*			63	*23*.*7*		
	**> 40 years**	49	*14*	0.57 (0.38–0.84)**	0.46 (0.29–0.72)**	42	*64*.*6*	0.99 (0.52–1.88)	**-**	39	*15*.*9*	0.61 (0.39–0.95)**	0.61 (0.39–0.95)**
**Religion Head Household**	**Orthodox**	128	*18*.*1*			99	*67*.*3*			102	*20*		
	**Others**	1	*20*	1.13 (0.13–10.19)	**-**	15	*48*.*4*	0.46 (0.21–0.99)**	0.95 (0.32–2.83)	0	*0*	**-**	**-**
**Number of people in household**	**< = 6 person**	73	*19*.*1*			83	*58*.*9*			55	*19*.*2*		
	**> 6 person**	56	*17*.*2*	0.88 (0.60–1.29)	**-**	31	*83*.*8*	3.61 (1.42–9.21)**	1.24 (0.39–3.91)	46	*20*.*1*	1.06 (0.68–1.63)	**-**
**Number of children in household**	**< = 3 children**	110	*21*.*2*			111	*66*.*1*			70	*19*		
	**> 3 children**	19	*9*.*9*	0.41 (0.24–0.69)**	0.47 (0.27–0.83)**	2	*22*.*2*	0.15 (0.03–0.73)**	0.11 (0.02–0.68)**	32	*21*.*8*	1.19 (0.74–1.90)	**-**
**SOCIO-ECONOMIC INDEXES**													
**Socio-Economic Index**	**Low**	37	*15*.*5*	1	1	19	*37*.*3*	1	1	30	*18*.*9*	1	
	**Medium**	51	*21*.*6*	1.50 (0.94–2.39)*	1.75 (1.06–2.89)**	27	*57*.*4*	2.27 (1.01–5.11)**	2.27 (0.87–5.95)*	39	*21*.*9*	1.21 (0.71–2.06)	**-**
	**High**	41	*17*.*3*	1.14 (0.70–1.85)	1.81 (1.05–3.11)**	68	*85*	9.54 (4.14–22.02)**	7.56 (2.79–20.47)**	33	*18*.*4*	0.97 (0.56–1.68)	**-**
**Socio-Educative Index**	**Low**	88	*22*.*6*	1	1	30	*39*.*5*	1	1	49	*17*.*4*	1	
	**Medium**	21	*12*.*8*	0.50 (0.30–0.84)**	0.47 (0.27–0.80)**	23	*88*.*5*	11.76 (3.24–42.62)**	5.66 (1.35–23.75)**	25	*22*.*3*	1.36 (0.79–2.34)	**-**
	**High**	20	*12*.*7*	0.50 (0.30–0.85)**	0.40 (0.22–0.73)**	61	*80*.*3*	6.24 (3.01–12.92)**	2.87 (1.16–7.08)**	28	*22*.*8*	1.40 (0.83–2.35)	**-**
**Community Endowment Index**	**Low**	65	*26*.*3*	1	1	29	*76*.*3*	1	1	42	*19*.*8*	1	
	**Medium**	27	*15*.*3*	0.50 (0.31–0.83)**	0.42(0.25–0.72)**	58	*54*.*7*	0.38 (0.16–0.87)**	0.35 (0.13–0.95)**	20	*19*	0.95 (0.53–1.72)	**-**
	**High**	37	*12*.*9*	0.41 (0.27–0.65)**	0.37 (0.23–0.60)**	27	*79*.*4*	1.20 (0.39–3.66)	1.24 (0.33–4.67)	40	*20*.*1*	1.02 (0.63–1.65)	**-**
**Goodness of fit**		**Nagelkerke R Square:0.139; Hosmer and Lemeshow Test p>0.005**				**Nagelkerke R Square: 0.457; Hosmer and Lemeshow Test p>0.05**				**Nagelkerke R Square:0.015; Hosmer and Lemeshow Test: N.S.**			

* p<0.10

**p<0.05. uOR: unadjusted odds ratio. aOR: adjusted odds ratio.

In the urban sites, the increased likelihood of consuming AFS was associated with the sex male of the head of the household, as well as increasing SES and SED scores. On the contrary, living in a household with more than 3 children, and an increasing CEI were inversely related to the child consumption of AFS.

## Discussion

This study shows that the diet of school-aged children living in Libo Kemkem and Fogera was mainly composed of basic staples, legumes, pulses and oil, and was more diverse in urban than in rural settings. The majority of the children only consumed foods from 3 or less different food groups the day before the survey and food from animal sources was a rare component in the children’s diet, especially in rural places.

In this study, various intermediate factors like sex and age of the head of the household were associated with low DDS and non-intake of ASF. The consumption of ASF was especially influenced by socio-economic-indexes. As far as we know, this is the first research to describe food intake by location and seasonality and to assess factors related to low DDS and ASF consumption in this area.

### Food consumption

As is common in developing countries, starchy staple foods were almost universally consumed in rural and urban settings, whereas flesh foods, eggs, and dairy products were less likely to have been consumed [30]. Ethiopian diet is mainly composed of cereals, tubers and root crops, pulses and oil seeds [8]. Surprisingly, the intake of traditional food items such as teff, *kotcho* and *bule* were more frequent in urban than in rural sites, although they are produced in rural areas [31]. According to a recent article, the growing demand for teff, particularly among Ethiopia’s expanding middle class, is already causing domestic prices for the grain to rise, meaning that small farmers are selling the bulk of their crop to urban consumers and the grain is no longer within reach of many poor rural Ethiopians [32]. In this study, consumption inequality by setting was predominantly caused by higher intake of pulses and legumes in rural areas and higher intake of meat and poultry in urban sites. After cereals, the second most important crop group in Ethiopia (in terms of acreage) are pulses [13]. Whereas cereals are rich in carbohydrates, pulses are rich in protein. In many African countries, pulses serve as an important meat substitute [24]. In Amhara region, pulses and legumes account for 14% of total food expenditures, the highest expenditure share when compared to the rest of Ethiopian regions. Moreover, only for pulses and legumes is the variance of income elasticity lower in rural areas than in urban areas [33].

Food supply of animal products has been previously reported to be very limited in this region, especially in rural areas, despite the existence of a large local livestock population [10]. No child consumed any fish during the previous 24 hours. This might be due to difficulties on fish preservation, or to some existing taboos which suggest that certain diseases can be spread through fish consumption [34]. At the time being, the United Nations Industrial Development Organization, through the Boku University of Vienna, is supporting the development of the fisheries sector in the Amhara region, including the overall postharvest management of fish and fish products [35].

The percentage of children who took any dairy the day before the survey was quite low. In Ethiopia, fresh milk is mainly given to small children and dairy are not so common in the traditional cuisine [36]. Campaigns to raise awareness and knowledge of the importance of milk and other dairy products for nutrition among school-aged children, their parents and teachers, have been recently developed through Ethiopia by the United States Agency for International Development (USAID) [37]. Therefore, some progress might be expected in the future. It is also remarkable the low intake of any kind of fruit by school-aged children (below 7%, in both settings). Finally, the relevant role of religious traditions in the Ethiopian diet cannot be ignored. The Ethiopian Orthodox Church, the main religion in the country, prescribes certain periods of fasting, which include all Wednesdays and Fridays as well as several long periods spread over the year. During fasting week days or periods only one meal per day is permitted, and the consumption of foods from animal origin is not allowed [38].

### Dietary diversity and related factors

Individual dietary diversity reflects how varied are the foods typically consumed by a person. On average, 78.9% of the children surveyed consumed 3 or less food groups the previous day to the survey. Despite international agreement on the important role of the diet diversity (DD) for infants and young children [39], there are currently no specific recommendations regarding the optimal number of foods or food groups that a school age child should consume [40]. There is, however, a consensus that higher DDS is desirable and that a larger number of foods or food groups can help meet daily requirements for a variety of nutrients [41].

The prevalence of low DDS was significantly higher in rural sites compared to the urban. This might be related to household food insecurity, as in the Amhara regional state, a significant proportion of rural households (45%) have been previously described as food insecure [31]. If the HH was female and younger than 40 years old, the child had greater chances to have a more diverse diet in rural settings. Parents play an important role in shaping their children’s eating habits by dictating variety and quantity of foods available to their child or through parents’ own food-related behaviors and parental feeding styles [42,43]. Although there is evidence showing that women-headed households are usually poorer than households headed by men in developing countries [44], our results are in line with a research carried out in Tanzania that showed that women who were the HH had higher DDS than those who were the wife of the HH [45]. Furthermore, a report from Bangladesh found out that in female headed households resources allocation favored diet and health in relation to other needs, impacting on the diet and nutrition indicators within the household [46].

Regarding urban areas, a higher socio-educative index was a gradual protective factor for low DDS in school-aged children. The educational level of the HH has been previously described to be a factor related to food insecurity in Addis Ababa city [47]. Furthermore, it has been suggested that educational level might be a better indicator of food security in Ethiopia than wealth and measures encompassing home and landownership [48].

### Intake of animal source foods and related factors

Only 18.1% of school-aged children living in rural communities consumed AFS the day before the survey, despite that 96.3% of the families owned animals [22]. The percentage of children taking ASF was higher in urban sites (64%), even if only 36.5% of the families reported to own animals in these settings [22]. For farm households in rural Ethiopia, livestock is an important asset that can provide regular income and be disposed of in hard times to provide a safety net [24], for which reason, these products might preferably be sold on the domestic market instead of locally consumed. Interestingly, consumption of products from the family´s own cattle and intake of ASF were both protective factors against stunting (aOR: 0.67; 95%CI: 0.46–0.96, and aOR: 0.51; 95%CI: 0.29–0.91, respectively) in these rural populations and not in the urban ones [21,22]. Consumption of meat was also previously described as a protective factors against zinc deficiency (aOR: 0.22; 95%CI: 0.06–0.83) in both settings in the same study population. Animal source foods such as meat, milk and eggs are deemed desirable as they offer high quality proteins, energy, are nutrient dense and an excellent source of micronutrients, such as iron and zinc [16].

In rural settings, the intake of ASF was higher in males than in females. This inequality has been previously described in adult population living in Southwest Ethiopia [49]. As expected, living in houses with more than three children was associated to lower ASF intake; households with a higher number of inhabitants experience more frequent periods of food shortage, especially if they are not economically productive [5]. School-aged children with higher SES were significantly more prompted to have consumed ASF in the last 24 hours. These findings agree with the contemporary nutritional transition: when income increases in low- and middle-income countries, an increase in animal food consumption occurs [50]. On the contrary, the percentage of children who took ASF was lower in those houses with medium and high SED and CEI when compared to the houses belonging to the lowest SED and CEI tertile. We lack of possible explanations for this. A higher CEI might be related to better access to market (and therefore, more options to sell ASF products), although this is only a weak hypothesis that needs to be further explored.

In urban areas, same association between consumption of ASF and number of children living in the house was found. In this case, higher socio-economic and socio-educative levels were associated with more frequent intake of ASF, compared to lower SES and SED levels, as it was expected. The association between educational level and food security has been widely reported by FAO [6,51]. For instance, educational activities within the Women-Focused Goat Development Program in the Highlands of Ethiopia have proven to increment ASF availability and access at household level [16]. Finally, children from the medium CEI tertile consumed ASF less frequently than those from the lowest tertile. As hypothesized before, better community assets might not necessarily mean better food consumption at the individual level.

### Seasonal variations in rural settings

In rural communities, the intake of vegetables non-rich in vitamin A, and pulses and legumes was higher in the post-harvest survey, while the consumption of fruit and vegetables rich in vitamin A, meat, poultry, and fats and oils was lower over the lean season, especially oils and fats. A strong seasonal periodicity for oilseeds has been previously seen in south central Ethiopia [52] and other zones belonging to Amhara region [53]. Another possible explanation might be the growing trend in the exports of oilseeds in the last decade [54]. Dairy products intake was 10 points higher in the post-harvest season. In this livelihood zone, the consumption of dairies increases during the wet season in November-January [10], when the production peak and farmers face bigger challenges marketing their dairies, as most regions experience a surplus, while animals are kept for later, when market situation improves and land preparation begins [36].

A lower DDS was found in the post-harvest season, with respect to the lean season in May. Seasonal vulnerability has been described in the area before [10,55]. What is less common is the use of DDS to assess these seasonal changes, despite that theoretical and empirical evidence suggest that DDS is an effective food and nutrition security indicator [56]. Nevertheless, some precautions are needed when interpreting seasonal changes in DDS: increased food availability may not necessarily imply higher dietary diversity, and not even increased food consumption, especially when trading activities and market constraints could be playing a role in this context, as in December and January the farmers must obtain cash for the next harvest [13,36].

While no differences were observed in the lean season by socioeconomic scores, in the post-harvest survey a higher socio-economic index was a gradually protective factor against low DDS. It is known that households with greater income also have the ability to purchase food products that are more diverse [51]. A higher SED was more common among those children with low DDS. Again, we lack of possible explanations for this association, given that most literature show the opposite relation [20,45,54].

With regards to consumption of ASF, the only significant related factor found in the post-harvest season was the age of the HH. This association also existed in the lean season: school-aged children with younger parents consumed ASF two times more frequently than those whose head of the household were older than 40 years. Moreover, a similar result was obtained for DDS (which was lower if the HH was older). This association might be related to the productivity level of the HH, meaning that it might be higher at younger age, although this assumption also needs to be tested.

### Limitations

The cross-sectional nature of the data does not allow examining causality in the relationship between low DDS, intake of ASF and associated factors. Another limitation is that children’s diet were analysed only qualitatively as quantity was not taken into account. Nevertheless, DDS has been validated as a useful tool to assess the likelihood of meeting micronutrient requirements Other limitations related to the study design have been described elsewhere [21,22,25].

## Conclusions

The findings of this study reveal that school-aged children diet in Libo Kemkem and Fogera districts lacked diversity, and that the intake of animal source foods was low, especially among rural girls. Overall, the area of residence seemed to be a predictor of children’s food habits, which highlights the need of stratifying the data in these type of contexts.

The age and sex of the head of the household, as well as other household characteristics, particularly socio-economic indexes, were found to be associated to ASF intake and low DDS. For the rural children, a lower DDS was found in the post-harvest season, while no difference was observed for the ASF intake, with respect to the lean season. At the light of these results, we believe that the promotion of dietary diversification strategies to improve children’s food consumption is needed in the study area. Interventions should target girls and households headed by male adults of older ages in the rural areas, and female headed households with low education level in the urban ones. We expect that findings documented by this study would assist policy makers plan and undertake regional initiatives.

## Supporting Information

S1 Table Distribution of selected characteristics of study children living in rural sites (May 2009 sample and December 2009 sub-sample), Libo Kemkem and Fogera, Ethiopia (DOCX)

S2 Table Variables included in the construction of each socio-economic index. (DOCX)
