# Supplementary material for: Low Dietary Diversity and Intake of Animal Source Foods among School Aged Children in Libo Kemkem and Fogera Districts, Ethiopia
Source: PLoS One. 2015 Jul 23;10(7):e0133435. doi: 10.1371/journal.pone.0133435 (PMC4512702; doi:10.1371/journal.pone.0133435)
Supplement: S1 Table — (DOCX) [file pone.0133435.s001.docx]

| **Supplementary Table 1. Distribution of selected characteristics of study children living in rural sites (May 2009 sample and December 2009 sub-sample), Libo Kemkem and Fogera, Ethiopia** | | | |
| --- | --- | --- | --- |
| **CHARACTERISTICS** | **Rural sample (May 09)** | **Rural Sub-sample (Dec 09)** | **p value** |
|  | **n=711** | **n=516** |  |
| **INDIVIDUAL CHARACTERISTICS** | | | |
| % female | 47.7 | 49.6 | p>0.05 |
| % ≥10 years old | 38.7 | 35.9 | p>0.05 |
| DDS high tertile | 4.0 | 3.7 | p>0.05 |
| DDS low tertile | 83.6 | 83.3 | p>0.05 |
| **HOUSEHOLD CHARACTERISTCS** | | | |
| Sex of household´s head(% female) | 7.3 | 7.2 | p>0.05 |
| Age of household´s head (>40 years old) | 49.7 | 47.9 | p>0.05 |
| Religion of household´s head (orthodox) | 99.3 | 99.0 | p>0.05 |
| Occupation of household´s head (unqualified) | 99.7 | 99.6 | p>0.05 |
| School years of household´s head (>4 years) | 16.5 | 17.6 | p>0.05 |
| School years of person in charge of food preparation (>4 years) | 1.3 | 1.4 | p>0.05 |
| Number of children in the household (>3 children) | 27.0 | 28.5 | p>0.05 |
| Number of people in the household (>3 people) | 46.0 | 44.5 | p>0.05 |
| Have domestic animals (yes) | 96.3 | 96.1 | p>0.05 |
| Own land (yes) | 97.6 | 97.7 | p>0.05 |
| Socio-economic index (low) | 33.5 | 30.8 | p>0.05 |
| Socio-educative index (low) | 54.9 | 54.5 | p>0.05 |
| Community endowment index (low) | 34.7 | 41.1 | p>0.05 |
