# Supplementary material for: Low Dietary Diversity and Intake of Animal Source Foods among School Aged Children in Libo Kemkem and Fogera Districts, Ethiopia
Source: PLoS One. 2015 Jul 23;10(7):e0133435. doi: 10.1371/journal.pone.0133435 (PMC4512702; doi:10.1371/journal.pone.0133435)
Supplement: S2 Table — (DOCX) [file pone.0133435.s002.docx]

| **Supplementary Table 2. Variables included in the construction of each socio-economic index** | | |
| --- | --- | --- |
| **INDEX** | | **VARIABLES** |
| **Socio-economic index** | Urban areas | The occupation of the head of the household |
|  |  | Electricity |
|  |  | Radio ownership |
|  |  | Flooring material |
|  | Rural areas | Land ownership |
|  |  | Annual crop production (in kilograms) |
|  |  | Livestock ownership |
|  |  | Flooring material |
| **Socio-educative index** | | Household head´s literacy |
|  |  | Years of schooling of the head of the household |
|  |  | Years of schooling of the person responsible for preparing the meals |
| **Community endowment index** | distance (in time) to: | The nearest health post |
|  |  | The nearest hospital, |
|  |  | The nearest paved road,, |
|  |  | The nearest permanent market , |
|  |  | The nearest establishment selling drugs |
|  |  | The nearest school. |
